# Structural fragility in digital public health communication

**DOI:** 10.1038/s44528-026-00037-y

**Published:** 2026-10-07

**Authors:** Miao Feng, Zizi Papacharissi, Chandler C. Carter, Mateusz Borowiecki, Simon Page, Ganna Kostygina, Natalie Wang, Yoonsang Kim, Brandon Sepulvado, Sherry L. Emery

**Affiliations:** 1 NORC at the University of Chicago, Chicago, IL USA; 2 Departments of Communication and Political Science, University of Illinois Chicago, Chicago, IL USA

**Keywords:** Complex networks, Complex networks, Cultural and media studies, Cultural and media studies, Health care, Health humanities, Medical humanities, Politics and international relations

## Abstract

**Background:**

Digital public health campaigns often report robust reach and engagement metrics, yet these metrics obscure deeper structural vulnerabilities. We define structural fragility as the declining capacity of actors to maintain network visibility in competitive digital communication environments, and assess it in Twitter/X discourse on U.S. e-cigarette prevention (2014–2023).

**Methods:**

Accounts were classified into five categories (Health, Commercial, Vape Community, Organic, Other). Longitudinal network analysis tracked three dimensions of structural fragility: positional (amplification and centrality), community (fragmentation and homophily), and core (k-core presence). Annual directed retweet networks were constructed, and modularity, in-degree, PageRank, eigenvector centrality, and k-core composition were computed. Regression models were adjusted for the number of participating accounts.

**Results:**

Here we show the network contracted from 36,204 accounts in 2014 to 800 in 2022, a 97.8% reduction. It fragmented into increasingly homophilous clusters (modularity 0.16 to 0.81; within-category retweeting 6.7% to 42.6%). Health accounts fell from 59.1% of the deepest k-core in 2014 to 3.8% in 2015, as Vape Community accounts rose to 78.8%. Per-capita amplification declined 95.7%, and Health PageRank share fell from 0.41 to 0.34, with amplification concentrated among a few accounts (eigenvector Gini ≥ 0.93). These declines persisted after adjustment for account numbers.

**Conclusions:**

Health actors remained active while losing structural position, a “bunker effect” of broadcasting without amplification. These findings challenge exposure-based evaluation models. Core embedding, within-category concentration, and PageRank share are computable from public network data and offer practical measures for campaign evaluation and platform transparency.

## Introduction

Digital platforms have become central to public health communication, offering new scale and reach to U.S. public health campaigns, including e-cigarette prevention campaigns^1^. Yet surface-level metrics such as reach, impressions, and engagement can mask structural vulnerabilities. Despite strong reach and engagement, many campaigns struggle to maintain narrative impact, with some facing overwhelming volumes of oppositional messaging^2^. This disconnect between visibility and influence signals a tension between public health goals and attention-driven platform economies.

This tension reflects a broader challenge for public-interest communication in digital environments, where accurate information, marketing, policy advocacy, and entertainment compete for visibility. Public health communication increasingly operates within ecosystems shaped by algorithmic curation^3^, opaque moderation^4^, and competing narratives, including those promoted by industries with conflicting interests. These forces represent commercial determinants of health (CDoH) that can shape the structural organization of digital discourse^5^. While reach and impressions remain important, they often obscure whether messages are repeatedly propagated by key amplifiers beyond their immediate followers. Evaluations relying solely on engagement metrics may therefore overlook the structural conditions under which authoritative public health voices lose network position^6,7^.

Despite advances in message design and audience targeting, public health research rarely examines the network structures that shape whether campaigns sustain influence in competitive digital environments. Evaluation models have historically operated on the assumption that institutional authority confers visibility, a framework inherited from broadcast-era paradigms^8^. Yet scholarship on attention economies and networked gatekeeping suggests that engagement-optimized platforms may disadvantage institutional actors accustomed to broadcast-era visibility^3,9^. Adjacent fields, such as political communication and media studies, have documented this structural misalignment^10,11^, but public health scholarship has less often extended beyond reach and engagement metrics toward structural analyses^12,13^.

We introduce the concept of structural fragility: a systematic erosion of the positional advantage held by health actors within communication networks, reflecting a decline in the collective capacity to sustain amplification and cross-community embedding in contested digital information environments. Unlike reduced participation or message failure, structural fragility describes a relational displacement in network position that unfolds even when institutional actors remain active and visible, operating at the level of network topology rather than message content. Structural fragility is distinguished from polarization, echo chambers, and fragmentation by its asymmetry: those constructs describe community-level separation without distinguishing among actor classes, whereas structural fragility diagnoses the erosion of a specific actor class’s capacity to remain embedded across communities.

To operationalize this concept, we measure three dimensions – community, positional, and core fragility – using longitudinal network analysis of retweet relationships in U.S. e-cigarette prevention discourse on Twitter/X from 2014 to 2023. This approach provides a structural lens for evaluating digital campaigns across their full lifecycle, including post-campaign dynamics that increasingly shape public health influence in evolving media and policy environments. E-cigarette prevention is a particularly informative case because youth e-cigarette use became a major public health concern during the study period: e-cigarettes became the most commonly used tobacco product among U.S. middle and high school students in 2014, high-school current use rose sharply in the late 2010s, peaked around 2019, and remained the most commonly used youth tobacco product in 2023 despite subsequent declines^14^. We treat e-cigarette prevention as a critical case^15,16^ of highly contested, commercially relevant public health communication. We offer a framework for understanding how public-interest communication more broadly contends with structurally constrained visibility in contemporary digital systems. We treat structural fragility as a mechanism-agnostic diagnosis: the framework identifies positional erosion at the level of network architecture without assuming which specific driver (algorithmic ranking, attention incentives, or actor strategy) produces it. Fragility is observed when institutional public-interest communicators lose cross-community embedding, amplification, or core presence in the diffusion network, operationalized here as the Health category (agencies, providers, researchers). We find that from 2014 to 2022, the retweet network surrounding these digital campaigns shifted from a globally integrated to a globally fragmented, yet locally concentrated, structure: communities became increasingly isolated from one another, while amplification within communities became more concentrated. This growth in fragility across community, positional, and core measures documents a persistent decline in the amplification and network position of Health actors, even as institutional accounts continued to tweet. Future health campaigns may therefore benefit from a network-aware stance, tailoring both campaign strategy and evaluation to message diffusion rather than exposure alone.

## Methods

### Study design, setting, and data sources

We conducted an observational longitudinal network study of retweet amplification in U.S. e-cigarette prevention discourse on Twitter/X from January 2014 through April 2023, when the platform was named Twitter (renamed X in July 2023); we use “Twitter/X” for the platform and retain the contemporaneous terms “tweet” and “retweet” for the content analyzed. Retweets were treated as diffusion ties to capture amplification dynamics, rather than conversational interactions^17^.

Public Twitter/X data were collected monthly using hundreds of keyword search rules for broad data collection on the topic of tobacco control, via the Historical PowerTrack and Academic Research APIs. These rules yielded millions of tweets per month, from which the campaign-relevant subset described below was extracted. The data were used in accordance with Twitter’s Terms of Service, and the study was classified as exempt under Category 4 (secondary research involving publicly available data) by the Institutional Review Board at NORC at the University of Chicago (approved July 12, 2023). We then developed a list of e-cigarette prevention campaigns that were active during the period of 2014–2023. We included one tobacco prevention campaign (Fresh Empire) targeting combustible products that appeared in tweets about e-cigarettes. Using a list of campaign-specific hashtags, institutional user handles, and supplemental keywords (Table 1), we extracted a subset of our Twitter/X data that matched prevention efforts active during the period.

**Table 1:** Inventory of Campaign Hashtags and Direct @ Handles Leveraged For Data Collection

Campaign name	Campaign region	Hashtag #1	Hashtag #2	Direct @ Handle
Still Blowing Smoke	California	#stillblowingsmoke		
The Vape Talk	Pennsylvania	#thevapetalk		
Facts Over Flavor	Arizona	#FactsOverFlavor		
Vape Free November	Colorado	#VapeFreeNovember		
I Just Didn’t Know	Kentucky	#IJustDidntKnow		@healthyky
Be Smart, Don’t Start	Vermont	#BeSmartDontStart		
The Dirty Truth	Delaware	#TheDirtyTruth		
The Real Deal on Vaping	Pima County	#TheRealDealOnVaping		
Vaping Truth	Illinois	#VapingTruth		
Vaping Equals	Kentucky	#VapingEquals	#WeAreJCPS	@JCPSKY
Flavors Hook Kids	California	#FlavorsHookKids		
behind the haze	Kentucky	#BehindTheHaze		
The Real Cost	National	#therealcost		@FDATobacco
The Fresh Empire	National	#freshempire		@freshempire

Records from January 2014–December 2019 were obtained via the Historical PowerTrack API; records from January 2020–April 2023 were obtained using the Academic Research API following a change in platform access. Partial year 2023 data (January–April) were retained to provide continuity in descriptive analysis but were excluded from statistical trend analyses.

To minimize off-topic content, we applied a campaign-specific filter composed of regular expressions and inclusion rules developed through iterative review. For example, tweets from institutional accounts such as @FDATobacco were retained only when at least one campaign hashtag was present. Inclusion criteria required at least one campaign-relevant retweet within a given year. Annual networks were therefore constructed from users appearing in the year-specific retweet edge list (as retweeters or retweeted accounts). Retweets involving accounts without a final classification were excluded, yielding 199,504 retweets for analysis. A data-flow diagram appears in Supplementary Figure 1.

### Account classification and human validation

To examine the roles of different actors, we classified all unique users in the analytic data into five mutually exclusive categories: Health (health-related organizations or individuals including public health agencies, healthcare providers, researchers, etc.), Commercial (organizations or brands promoting or selling tobacco products), Vape Community (organizations or individuals promoting e-cigarette use or pro-e-cigarette policies/agendas), Organic (individual users without public or institutional affiliations), and Other (accounts not classified in the preceding categories, such as media aggregators or organizations without sufficient information for attribution).

User classification followed a two-step process combining large language model (LLM)-based classification with human validation. First, we used GPT-4o on Microsoft Azure to classify all unique users in the analytic data into five mutually exclusive categories, using a structured prompt (full prompt available in Supplementary Information). Since Twitter/X display names and usernames are non-unique and mutable, we classified each unique user ID based on the observation with the highest follower count, aiming to select the most complete, most recent metadata for that account. Account classifications were merged with the annual retweet-network data for category-specific analyses.

In the full dataset of unique user IDs, Organic Users accounted for 62.2% of accounts, while Health Actors (2.3%), Vape Community (2.0%), and Commercial accounts (1.3%) were relatively rare. To ensure adequate representation of these low-prevalence categories, the human validation sample was stratified by model-assigned account type, resulting in over-sampling of rarer classes and under-sampling of more prevalent ones.

To validate LLM-based classifications, we first drew a random sample of 160 accounts and one example tweet per account for double-coding by two trained coders to assess interrater reliability. Coders assigned account-level labels using a grounded theory–informed codebook. Reliability was high for the Health, Commercial, Vape Community, and Other categories (AC1 > 0.80), and moderate for the Organic category (AC1 = 0.76)^18^. We then drew a random sample of 200 unique Twitter/X users, stratified by user category (40 per category) to ensure balance across account categories. Coders independently reviewed each unique user and assigned account-level labels; disagreements were adjudicated by a senior coder, and the adjudicated labels served as the reference standard for evaluating GPT-4o classification performance. Human coders could consult up to ten recent tweets to resolve ambiguous cases, whereas the classifier received a single example tweet, so reported performance reflects agreement against a more fully informed standard.

Additional classification details, class-specific precision, recall, and F1 scores, the labeling codebook used by human coders, and the full structured prompt and output schema are provided in the Supplementary Information.

Across categories, the classifier achieved a F1 score of 0.86 (both weighted and macro). Performance was highest for Health accounts (F1 = 0.95) and Organic accounts (F1 = 0.86), with slightly lower performance for Commercial (F1 = 0.85), Other accounts (F1 = 0.83), and Vape Community (F1 = 0.80).

### Network construction and measures

The choice of retweets as the unit of analysis is deliberate because this study focuses on diffusion-network position rather than all forms of campaign communication. Retweets are a central diffusion mechanism on Twitter/X: they propagate content across follower networks, increase repeated exposure, and generate cascading visibility^19,20^. We therefore interpret retweet ties as content-mediated amplification events. The resulting retweet topology is an outcome of users’ sharing decisions, not a structure independent of content. Health actors communicate through multiple interaction types, including original tweets, replies, quote tweets, and retweets; however, these interaction types serve different communicative functions and generate different network structures. Because structural fragility concerns whether public health actors remain embedded in the pathways through which campaign messages are amplified, retweets provide an appropriate operationalization of diffusion-network position. This focus also makes the scope of inference explicit: the analysis evaluates Health actors’ position in retweet-based diffusion networks rather than their total communicative activity on Twitter/X.

For each calendar year (2014–2023), we constructed a directed, weighted retweet graph from the campaign-relevant corpus. Each node represents a unique user who retweeted or was retweeted at least once during that year. A directed edge from user A to B indicates that A retweeted B; multiple retweets within a year were collapsed into a single weighted edge with dyad-year weight equal to the aggregated number of retweets between the pair. We did not impose a minimum degree threshold; node-level metrics were computed for all accounts present in each annual retweet graph.

Measures were bound to specific graph types. Density was computed on the directed retweet graph. Modularity (subgroup separation; 0–1 scale, with higher values indicating greater community segregation) was computed on the undirected weighted projection of the retweet graph after removing self-loops^21^. Eigenvector centrality was computed on the undirected weighted projection; when the projected graph was disconnected, eigenvector centrality was computed on the largest connected component (LCC), with nodes outside the LCC assigned a value of 0. The LCC ratio was defined as the proportion of nodes in the LCC of the undirected projection. PageRank was computed on the full directed, weighted retweet network with damping factor of 0.85 and uniform redistribution of dangling mass; category-level PageRank mass was summarized as the sum of PageRank values within each category. K-core analyses were conducted on the undirected projection. Attribute assortativity by category was computed on the unweighted undirected projection after removing self-loops, whereas within-category retweet share was computed on weighted retweet events. The two homophily indicators therefore capture related but distinct aspects of network segregation.

Primary indicators were: (1) core embedding, operationalized as category shares of deepest k-core membership; (2) network visibility, captured by category-level PageRank mass and in-strength totals (and their corresponding category shares); and (3) community integration, assessed using modularity and the LCC ratio. Secondary indicators included in-degree per 1,000 category members (per-capita amplification efficiency)^22^, assortativity by category (the tendency for similar nodes to connect in the network; −1 indicating disassortative mixing; +1 indicating assortative mixing)^23^, within-category retweet share (the proportion of retweets occurring between users of the same category), amplification probability (the proportion of accounts in each category retweeted by at least one other account; reported in Supplementary Tables 5–9), and eigenvector concentration (the extent to which centrality is concentrated among a small subset of nodes). Additional results for secondary indicators are provided in the Supplementary Information.

### Operationalization of structural fragility and the bunker effect

We conceptualized structural fragility as a declining capacity of Health actors to sustain amplification and cross-community embedding in the diffusion network. Positional fragility was assessed via in-strength, in-degree, amplification probability (reported in Supplementary Tables 5–9), eigenvector centrality, PageRank, and per-capita amplification efficiency. Community fragility was assessed via modularity, LCC ratio, assortativity by category, and within-category retweet share. Core fragility was assessed via deepest k-core size and composition.

A year was classified as “bunker consistent” relative to the 2014–2015 baseline when modularity increased, the LCC ratio decreased, and within-category retweet concentration and assortativity increased, indicating a contracted, internally reinforcing topology that restricts cross-community diffusion.

### Statistics and reproducibility

Primary network measures were computed in Python 3.14 using NetworkX 3.6.1, with main-analysis community partitions identified using the Louvain algorithm implemented in python-louvain 0.16 at default resolution^24^. The degree-preserving null analysis reported in Supplementary Table 4 used python-igraph 1.0.0 for graph rewiring and multilevel community detection, applying the same implementation to observed and randomized networks.

Annual trends (2014–2022) were evaluated using Mann–Kendall tests with tie-adjusted variance and the standard continuity correction, together with Theil–Sen estimates of median annual slope (Supplementary Table 1). For the two community-fragility indicators, modularity and largest connected component ratio, we additionally estimated ordinary least squares models with classical standard errors. Activity-adjusted analyses of Health amplification used the same estimator, with the annual number of participating Health accounts included as an additional covariate. Because adjacent annual network measures may be serially dependent, neither the classical standard errors nor the Mann–Kendall p-values fully account for temporal dependence, and uncertainty may be understated under positive serial correlation. Trend tests were interpreted descriptively, and all tests were two-sided. Abrupt changes were assessed through visual inspection and year-to-year contrasts rather than assumed linearity.

To evaluate whether observed patterns exceeded chance expectations, we conducted two null-model analyses. The attribute-permutation null held the observed yearly topology fixed and randomly permuted actor-category labels across nodes 1,000 times. This preserves network size, edge structure, and the yearly number of nodes in each category, and was used to evaluate category assortativity, within-category retweet share, Health and Vape Community shares of deepest k-core membership, Health share of PageRank mass, and Health share of eigenvector mass. The degree-preserving configuration-model null randomized the undirected weighted projection using degree-preserving edge rewiring with 500 randomizations per year; the observed edge-weight multiset was reassigned to rewired edges. This null preserves the unweighted degree sequence and the overall weight distribution, but not each node’s weighted strength. It was used to evaluate Louvain modularity, LCC ratio, and deepest k-core depth relative to degree-matched random networks. Empirical p-values are two-sided and reported with observed values, null means, 95% null intervals, and z-scores in Supplementary Tables 3 and 4.

Quality checks verified that category-level PageRank mass summed to 1.0 and that deepest k-core category shares summed to 100% within each year. Code and data availability are described in the respective statements below.

## Results

We analyzed retweet networks from 2014 through 2023, performing statistical trend tests on complete years (2014–2022) to evaluate the evolution of structural fragility across three dimensions. Values for 2023 (first four months) are reported descriptively where relevant.

We report each fragility dimension in turn: community fragility (modularity and LCC ratio), positional fragility (amplification, PageRank, and eigenvector centrality), and core fragility (deepest k-core composition).

### Visualizing structural fragility: network reconfiguration, 2014–2022

**Fig. 1:**
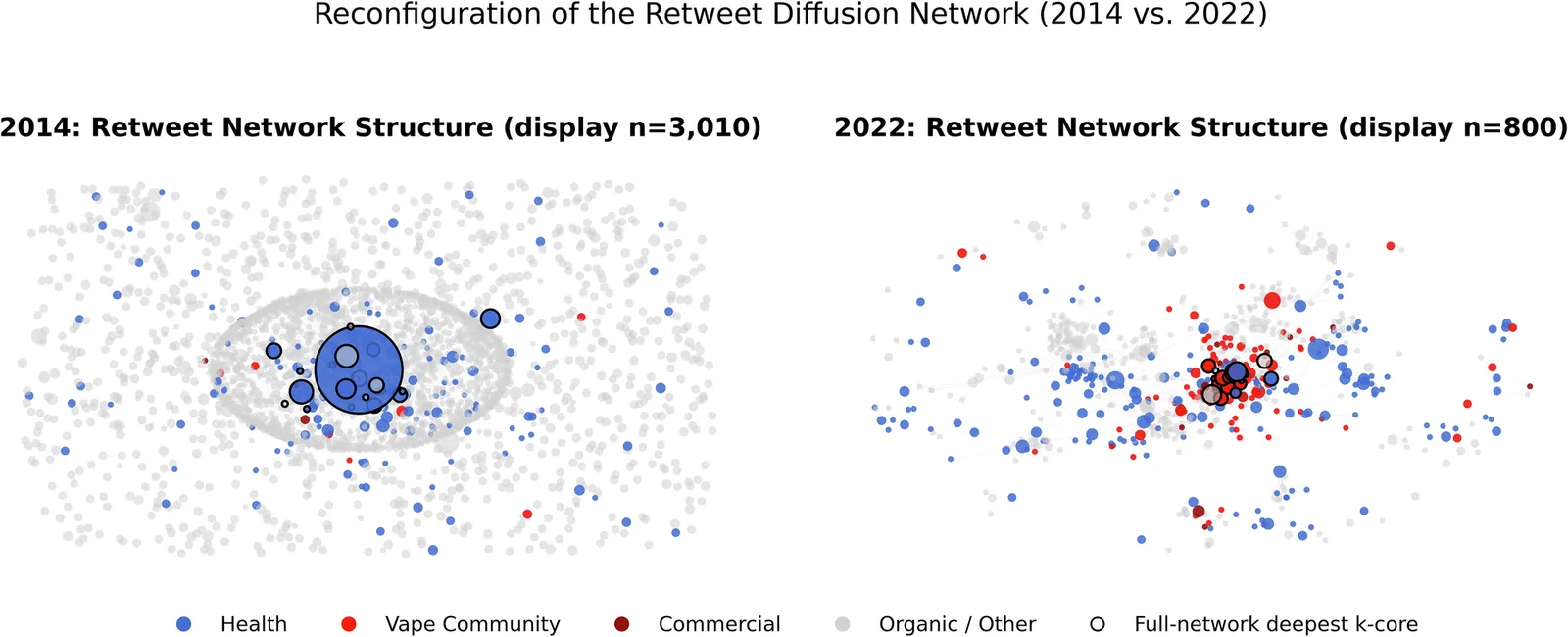
Visualizing structural fragility: Network reconfiguration, 2014–2022. Annual retweet networks of U.S. e-cigarette prevention discourse are shown as undirected projections for visualization. Nodes are sized by in‑strength (retweets received); Health, Vape Community, and Commercial accounts are shown in distinct colors, while Organic and Other accounts are de-emphasized in gray. Black outlines identify members of the deepest k-core computed on the full annual undirected projection. For legibility, the 2014 panel shows the 3000 highest-in-strength nodes plus any full-network deepest-core members omitted by that display cutoff; the 2022 panel shows all 800 nodes. Retweet frequency was used as edge weight in the underlying network analyses; displayed edge widths are fixed for legibility.

Figure 1 illustrates the reconfiguration of the retweet diffusion network over the study period. In 2014, Health actors (blue) occupied a centralized position within a dense core. By 2022, the network was smaller and more fragmented, with Vape Community nodes (red) concentrated in the core and Health nodes dispersed toward the periphery. Subsequent sections quantify these structural shifts using modularity and LCC (community fragility), PageRank/eigenvector (positional fragility), and k‑core composition (core fragility).

### Network-Level Participation Decline

The number of active users tweeting about e-cigarette prevention campaigns declined from 36,204 in 2014 to 800 in 2022, a 97.8% reduction (Table 2). Directed retweet edges decreased from 35,657 to 984 over the same complete-year period, and aggregate in-strength fell from 42,427 to 1,285. In the partial 2023 window (January–April only), 122 users remained active; 2023 is therefore reported descriptively and excluded from trend tests. Across the full analytical dataset, user composition comprised Organic (62.2%), Other (32.2%), Health (2.3%), Vape Community (2.0%), and Commercial accounts (1.3%).

**Table 2:** Overall descriptive network statistics, 2014–2023*

	2014	2015	2016	2017	2018	2019	2020	2021	2022	2023*
Nodes	36,204	21,438	21,180	21,385	12,925	5723	1440	1212	800	122
Edges	35,657	26,306	27,739	22,817	13,092	5837	1567	1501	984	114
Density (directed)	0.000027	0.000057	0.000062	0.000050	0.000078	0.000178	0.000756	0.001023	0.001539	0.007723
Modularity	0.162	0.662	0.686	0.530	0.622	0.785	0.884	0.771	0.808	0.836
LCC Ratio	0.938	0.951	0.935	0.897	0.895	0.819	0.632	0.677	0.501	0.172
Deepest k-core Size	22	52	52	27	12	119	27	27	22	14
Assortativity	−0.48	−0.16	−0.01	−0.27	−0.33	−0.09	0.17	0.10	0.26	0.32
Within-category retweet share (%)	6.7	25.4	29.9	17.0	15.3	25.8	42.2	38.4	42.6	51.7

*LCC* Largest Connected Component. Density was computed on the directed retweet network. Modularity was computed on the undirected weighted projection after removing self-loops; LCC ratio and k-core measures were computed on the corresponding undirected topology. Attribute assortativity was computed on the unweighted undirected projection after removing self-loops; within-category retweet share was computed on weighted retweet events. *January 2023 to April 2023.

### Community fragility: fragmentation and homophily

**Fig. 2:**
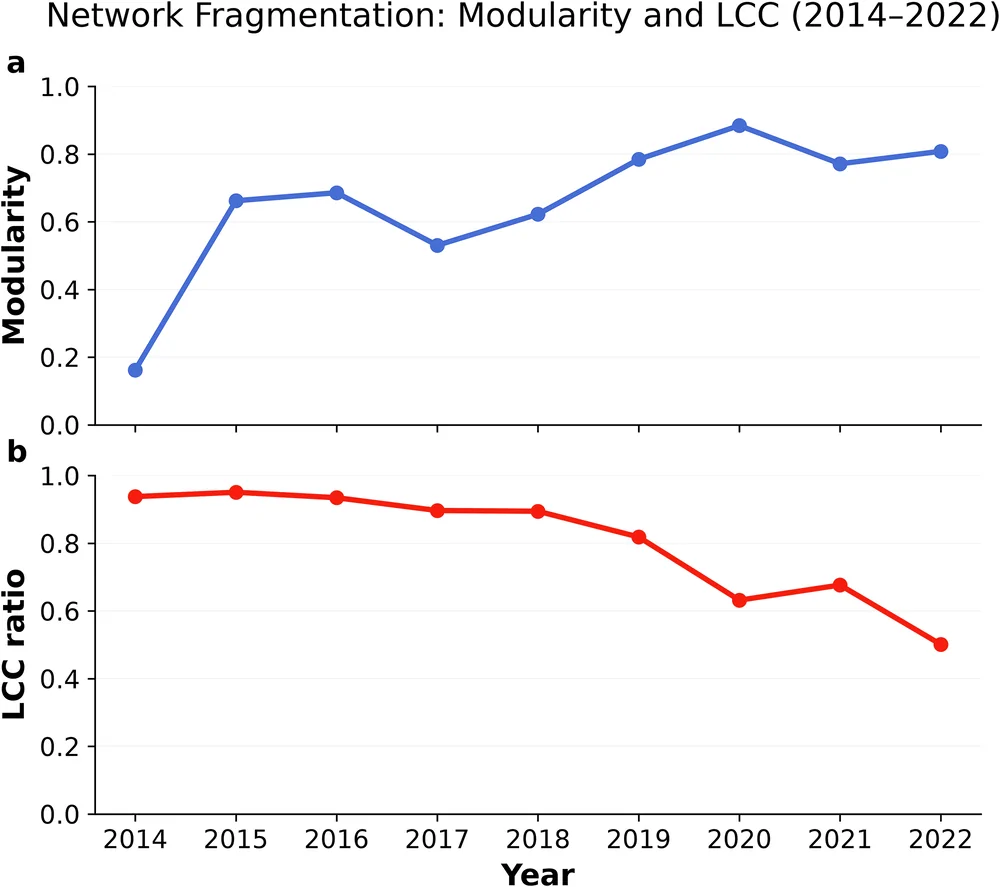
Network fragmentation: modularity and LCC (2014–2022). **a** Shows modularity computed on the undirected, weighted projection of the yearly retweet graph (self-loops removed); **b** Shows the LCC ratio, defined as the share of nodes in the largest connected component of the undirected projection. Points represent complete years (2014–2022) used in trend analyses.

Network-level connectivity declined substantially over time. As shown in Fig. 2, the LCC ratio decreased from 93.8% in 2014 to 50.1% in 2022 (β = −0.054, 95% CI [−0.076, −0.033], *p* < 0.001, R² = 0.835), and fell to 17.2% in the partial 2023 period (descriptive), signifying a contraction of the global conversation. Simultaneously, Louvain modularity increased from 0.16 to 0.81 (β = 0.059, 95% CI [0.014, 0.105], *p* = 0.018, R² = 0.577) and reached 0.84 in the partial 2023 period (descriptive). These shifts indicate a transition from a relatively unified communication system to a fragmented environment of separated network clusters.

**Fig. 3:**
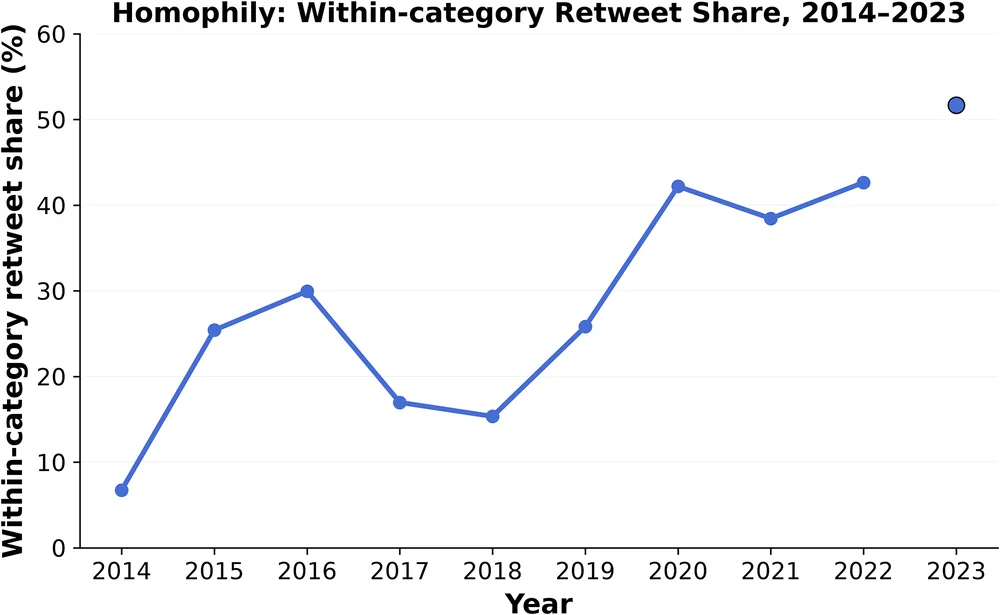
Homophily: Within-category Retweet Share, 2014–2023 (descriptive). Within-category retweet share by year, defined as the proportion of retweets in which the retweeter and the original author belong to the same account category (Health, Vape Community, Commercial, Organic, Other). Values are computed annually on the full directed, weighted retweet graph (edges weighted by retweet counts). Points for 2014–2022 represent complete years used in analyses; the 2023 value (January–April) is shown as a standalone descriptive point and is excluded from trend tests.

Interaction patterns became increasingly insular by actor category. Within-category retweeting, displayed in Fig. 3, increased from 6.7% in 2014 to 42.6% in 2022, reaching 51.7% in the partial 2023 period, indicating a shift toward homophilous interaction patterns among actor categories. Similarly, attribute assortativity rose from −0.48 in 2014 to 0.26 in 2022 and 0.32 in the partial 2023 period, consistent with stronger within-group ties and increasing community segregation. While directed-graph density rose from 0.000027 to 0.001539 (2022) and to 0.007723 in 2023 (descriptive), this metric is interpreted alongside assortativity and modularity because density is highly sensitive to the contracting network size. Collectively, these patterns suggest a hardening of category boundaries, where narratives are increasingly confined to internal reinforcement rather than cross-community diffusion (Fig. 3).

Applying the bunker-consistent criterion defined in the Methods, six of the seven post-baseline years (2016, 2017, 2019, 2020, 2021, and 2022) met all four conditions relative to the 2014–2015 baseline. The exception was 2018, when assortativity ( − 0.33) and within-category retweet share (15.3%) both fell below baseline values.

### Positional fragility: amplification and centrality

**Fig. 4:**
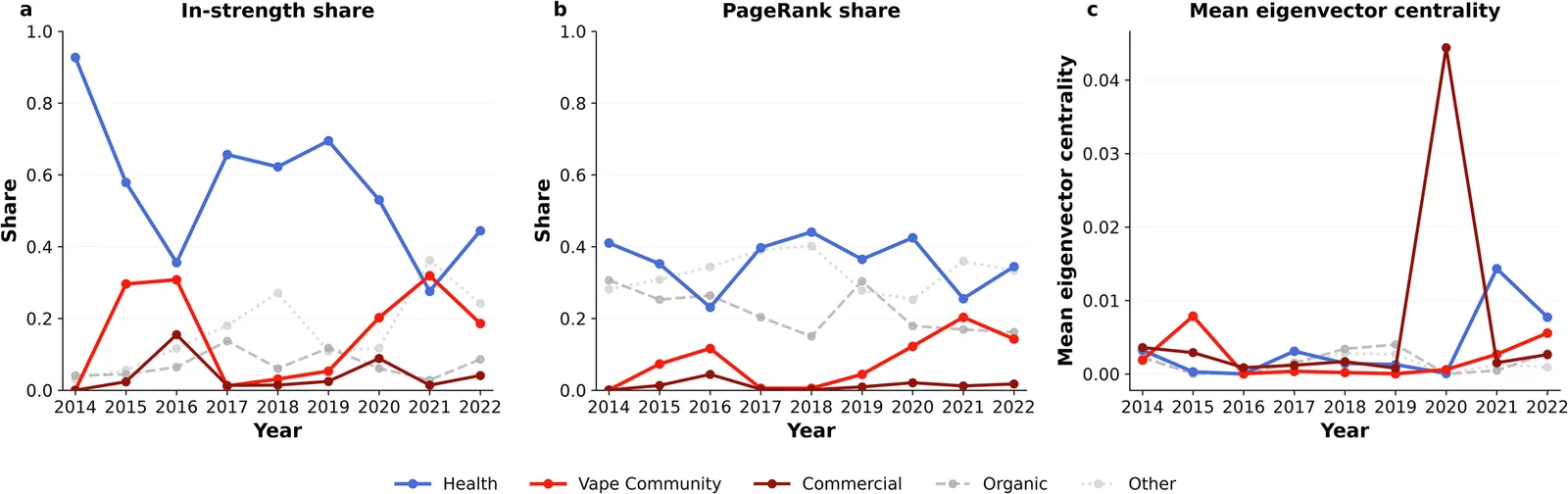
Amplification (In-Strength Share), PageRank Share, and Eigenvector Mean by Category, 2014–2022. **a** In-strength share by category. **b** PageRank share by category computed on the full directed, weighted graph (damping = 0.85). **c** Mean eigenvector centrality by category computed on the undirected, weighted projection. Values shown correspond to complete years only (2014–2022). Eigenvector centrality was computed on the undirected projection; when disconnected, values were computed on the LCC and nodes outside the LCC were assigned 0.

Health actors’ retweet-based amplification declined sharply. Per-capita amplification efficiency, measured as in-degree (the number of distinct accounts retweeting a category’s members) per 1000 category members, declined from 45,380 in 2014 to 1957 in 2022, a 95.7% decrease. Between 2018 and 2019, the number of active Health accounts increased from 569 to 600, while mean in-degree declined from 16.27 to 6.59. Category-level shares of total in-strength are shown in Fig. 4 (left).

Health eigenvector centrality was highly skewed within the Health category throughout the study period (Supplementary Table 1). The Health eigenvector Gini coefficient was 0.79 in 2014 and ranged from 0.93 to 1.00 in subsequent years. This concentration was present from the earliest study year and did not increase monotonically over 2014–2022 (eigenvector Gini: Mann–Kendall *p* = 0.47; Supplementary Table 1). Because this skewness makes the mean sensitive to a small number of high-centrality accounts, we do not use mean eigenvector centrality as a primary indicator of positional fragility.

Health in-strength and PageRank top-1% share declined over 2014–2022 (Mann–Kendall z = -2.81, *p* = 0.005 and z = -2.40, *p* = 0.017, respectively). Category-level PageRank also shifted descriptively: Health PageRank share declined from 0.41 in 2014 to 0.34 in 2022, while Vape Community share increased from approximately 0.00 to 0.14 (damping = 0.85). Mann–Kendall trend tests for aggregate Health PageRank mass and Health eigenvector mass provided no evidence of monotonic change (Supplementary Table 1). Overall, positional indicators show a sharp decline in retweet-based amplification, while recursive centrality measures show more heterogeneous and non-monotonic patterns.

### Core fragility: K-core composition

**Fig. 5:**
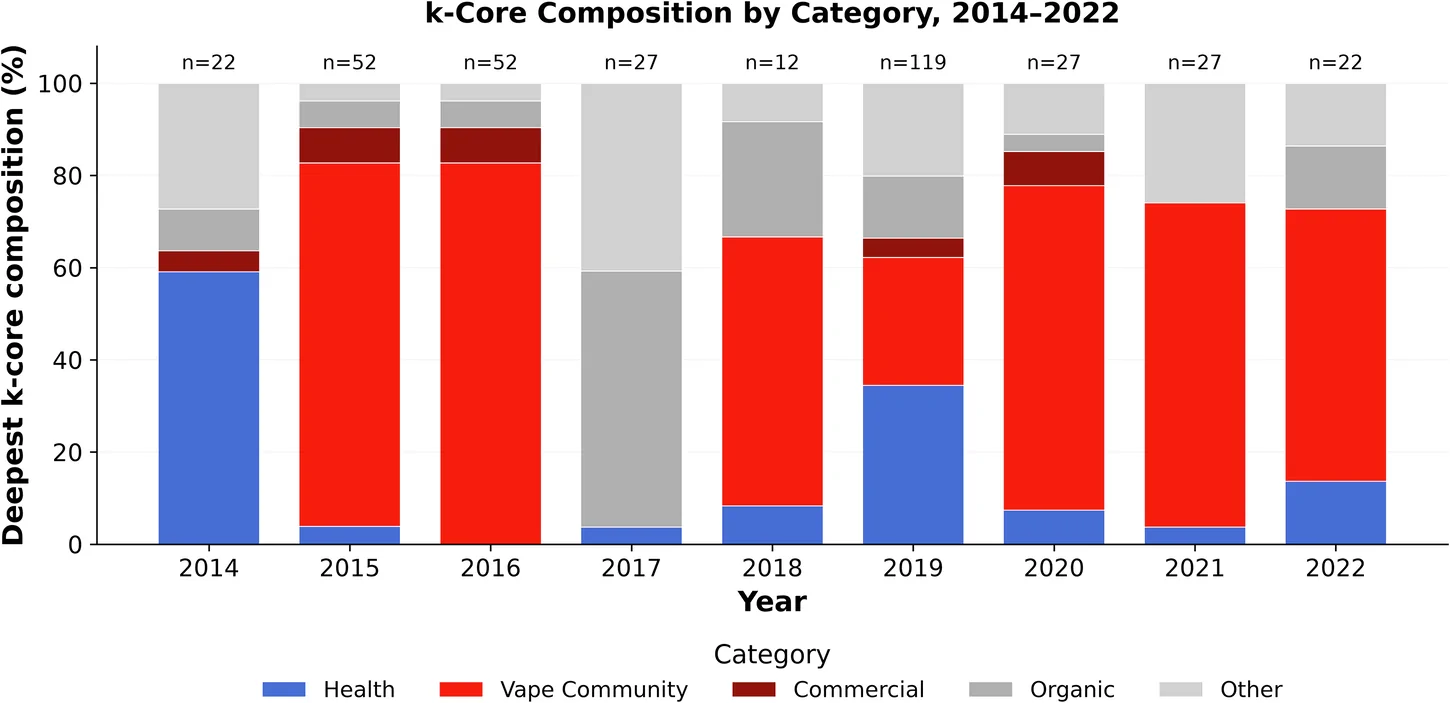
k-Core Composition by Category, 2014–2022. Shares of deepest k-core membership by category, computed on the undirected projection of each full annual retweet network. Stacked bars show annual core composition for complete years (2014–2022); labels above bars indicate the number of actors in each year’s deepest k-core.

Health actors’ presence in the network core decreased sharply. K-core analysis (on the undirected projection of the yearly retweet network) showed Health’s share falling from 59.1% (13/22 nodes) in 2014 to 3.8% (2/52) in 2015, while Vape Community rose to 78.8% (41/52) (Fig. 5). By 2022, Vape Community held 59.1% of core positions and Health 13.6%. Mann–Kendall tests provided no evidence of monotonic trends in k-core shares (Health, *p* = 1.00; Vape Community, *p* = 0.75). The largest change in Health core share occurred between 2014 and 2015, consistent with an abrupt reconfiguration rather than gradual erosion. Vape Community presence thereafter was recurrent but discontinuous, constituting the largest core category in six of the eight subsequent years.

### Robustness to activity intensity

To assess whether declining amplification reflected changes in campaign activity, we examined Health actor participation alongside amplification metrics. Health accounts remained numerous in mid-period years, yet amplification continued to decline: mean in-degree decreased from 16.27 in 2018 to 6.59 in 2019, and mean in-strength decreased from 19.67 to 8.87 over the same interval. Correlations between the number of Health accounts and amplification were weak across complete years (Pearson’s r = 0.10 for in-degree; r = 0.02 for in-strength; *n* = 9 annual observations). In category-year regression models (*n* = 9 annual observations) controlling for the number of participating Health accounts, the estimated year effects on Health amplification remained strongly negative (a decline of approximately 10.1 in-degree points per year, 95% CI [−13.1, −7.1], *p* < 0.001; and 13.3 in-strength points per year, 95% CI [− 18.4, −8.2], p < 0.001). These results suggest that the erosion of amplification is not explained by reduced campaign activity alone.

To assess whether the structural patterns depend on the single-retweet inclusion criterion, we recomputed the primary indicators under minimum activity thresholds of ≥2 and ≥5 campaign-relevant retweets per user-year (Supplementary Table 2). The abrupt core displacement was unchanged across thresholds. Comparing 2014 with 2016, Health actors’ share of the deepest k-core declined from 59.1% to 0.0%, while Vape Community actors’ share increased from 0.0% to 82.7%; the largest single-year change occurred between 2014 and 2015. The community-fragility trajectory was also stable, with rising modularity and declining LCC ratio observed under stricter inclusion rules. The Organic node share was larger under the single-retweet threshold and declined under stricter cutoffs, for example, from 59.7% to 39.5% in 2016 and from 35.6% to 9.3% in 2020. However, the core and community findings remained unchanged when low-activity accounts were excluded, indicating that the structural conclusions do not depend on the larger Organic category.

### Null-model analysis

We conducted two null-model analyses to assess whether the observed category positions and global topology differed from expectations under the specified randomization schemes. First, under the attribute-permutation null, Health actors’ deepest-core share was above chance expectation in 2014 and 2019, while Vape Community actors’ deepest-core share was above chance expectation in 2015–2016 and 2018–2022 (Supplementary Table 3). Category assortativity was significantly below null expectation in 2014, 2017, and 2018, indistinguishable from null in 2015, 2016, and 2019, and significantly above null expectations in 2020–2022. Retweeting was therefore more cross-category than chance early in the study period and more within-category than chance by its end. Second, under the degree-preserving configuration-model null, observed Louvain modularity was above degree-matched null expectations in most years after 2014, and the LCC ratio fell below degree-matched expectations in the later years (Supplementary Table 4).

Together, these analyses indicate that the observed category positions and global topology are inconsistent with the corresponding randomization models. This suggests that structural fragility reflects more than a simple decline in topic popularity or a mechanical reduction in the number of Health actors, although topic contraction remains an important contextual feature of the case.

## Discussion

Across a decade of digital e-cigarette campaign discourse on Twitter/X, the retweet network transitioned from a relatively integrated system to a globally fragmented yet locally concentrated structure, with institutional actors aiming to discourage e-cigarette use losing much of their presence in the network core and experiencing reduced diffusion across the broader network. Communities became increasingly disconnected from one another, while influence within surviving clusters became more centralized. We interpret these patterns as structural constraints on visibility rather than as direct evidence of platform-level causal mechanisms. We document three forms of structural fragility, each with a distinct temporal signature. *Community fragility* emerged gradually, evidenced by rising modularity, declining LCC ratio, and increasing within-category retweeting. These patterns are consistent with a “bunker effect,” in which homophily and the erosion of cross-cutting ties produce structural contraction, limiting cross-community diffusion even when broadcasting continues. *Core fragility* appeared as an abrupt 2014–2015 displacement in k-core membership, when Vape Community actors overtook Health actors and thereafter recurred in the diffusion core as the largest category in six of the eight subsequent years. *Positional fragility* manifested less as a smooth decline in all centrality measures than as the collapse of early retweet-based amplification: per-capita amplification fell by 95.7%, and Health in-strength and PageRank top-1% shares declined. These patterns should not be interpreted as equivalent monotonic trends (Supplementary Table 1).

The asymmetry noted above has an analytic consequence: community-level fragmentation and actor-specific displacement are independent. A network can be highly modular without any actor class losing positional advantage, and an actor class can lose core embedding in a network that is only moderately modular. The present data illustrate this: modularity and category assortativity both rise (community fragility), but the core displacement is category-specific (Health actors displaced, Vape Community ascendant), a pattern not captured by symmetric fragmentation measures. The framework uses modularity as one input but interprets it asymmetrically through the lens of which actors are confined and which benefit from the emerging structure.

The early k‑core displacement and subsequent rise in Vape Community PageRank share describe a structural reconfiguration in which Health actors’ positional advantage eroded while oppositional accounts ascended to densely connected network strata. These patterns are consistent with platform architectures that allocate visibility through recursive, engagement-sensitive processes, suggesting a foundational structural misalignment in which broadcast-oriented communication may be systematically disadvantaged relative to engagement-optimized content. Our observational design does not permit causal attribution to specific algorithms, but the positional decline itself is empirically robust across the study period.

More broadly, our findings are consistent with a digital extension of the commercial determinants of health (CDoH) framework. Traditional CDoH scholarship extensively documents how corporate entities utilize political and public relations avenues to shape health outcomes^5^. However, the manifestation of CDoH within algorithmic architectures remains underexplored. While our classifier distinguishes between explicit Commercial entities and the Vape Community, both categories appeared within the deepest diffusion core in four of the nine study years. This shared core presence is consistent with structural alignment but does not establish coordination, and may help account for how CDoH-aligned interests occupy advantageous positions in platform diffusion architectures. The mechanisms producing this shared core presence cannot be determined from topology alone.

This interpretation is reinforced by prior campaign surveillance showing that oppositional amplification is often highly concentrated. In comparative surveillance of federal and regional campaigns, opposition was concentrated among a small subset of users, with the top 10% generating 57.9% of opposition retweets^25^. The present study documents a parallel structural pattern where Vape Community accounts are disproportionately represented in the deepest k-cores across most study years. While concentration is a ubiquitous feature of scale-free social networks and could potentially reflect organic power-law dynamics, the specific displacement of Health actors is consistent with an architecture-contingent vulnerability. Cross-platform comparative research is consistent with this view: Twitter/X’s structural features can facilitate retweet-based amplification by relatively concentrated advocacy and commercially affiliated clusters, contrasting with more decentralized and entertainment-oriented oppositional dynamics on platforms like TikTok^26^.

Our results also align with complex contagion theory^27^, which emphasizes that the adoption of health behaviors and norms often requires repeated exposures within clustered social contexts. Importantly, such clustering facilitates diffusion only when communities remain connected by sufficient bridging ties that allow exposures to accumulate across groups. In the fragile networks we observe, this condition no longer holds: the collapse of LCC and the erosion of cross-cutting ties isolate clusters from one another, producing a “bunker effect” in which reinforcement occurs locally but fails to translate into broader diffusion. Under these conditions, complex contagions may become structurally constrained rather than accelerated.

Network-aware strategies could therefore prioritize rebuilding bridging pathways through partners with cross-community reach, strategically placing messages through core-adjacent intermediaries with high recursive visibility, and monitoring core embedding and PageRank shares alongside traditional reach metrics. Evaluation should complement exposure and engagement statistics with network‑based indicators that track whether evidence‑based content remains embedded in the communication core where diffusion occurs. Reliance on reach-based metrics alone risks overlooking persistent structural exclusion that may coexist with stable or even high levels of posting activity. Practical indicators may include core embedding, measured as the share of k-core membership attributable to Health actors; recursive visibility, captured by category PageRank shares on the full yearly graph; and community integration, assessed through modularity, attribute assortativity, and the share of cross-community edges. Strategically, public health agencies should coordinate dissemination across platforms, build partnerships with high‑visibility intermediaries, and experiment with influencer- and community‑based models to reinsert institutional narratives into fragmented networks. These approaches emphasize re-embedding health content within diffusion pathways rather than simply increasing message volume.

Policy should extend beyond content moderation to infrastructural regulation. As visibility is increasingly allocated through networked and algorithmic processes, governance mechanisms will need to address how public-interest content is positioned within communication systems. Minimum transparency standards, such as aggregate visibility distributions for public-interest accounts, ranked exposure reporting, and audit mechanisms, could enable routine monitoring of core embedding and oppositional displacement and support more equitable conditions for evidence-based health communication.

Several methodological limitations temper these findings. First, our framework diagnoses structural position at the level of network topology and does not assess content-level moderators. Because messaging strategy, content quality, and framing were not held constant across campaigns, we cannot determine whether these factors mitigate or accelerate structural fragility. Our prior mixed-methods work has characterized framing and rhetoric in this discourse^2,25,26^, whereas the present study addresses network position. Second, the observational design does not permit causal attribution to specific algorithmic mechanisms, platform architectures, or actor strategies. Third, the analysis models retweet ties and therefore does not capture non-retweet activity, including replies, quote tweets, or conversational sentiment. Because the corpus was identified through campaign hashtags and handles, it captures campaign-centered discourse but may omit e-cigarette discussion that did not use those identifiers, and it tracks the activity of specific campaigns as well as the structure of the surrounding discourse. Fourth, the networks contracted substantially over the 2014–2022 analytic period, and year was strongly associated with log node count (r = −0.95). Modularity, density, and the LCC ratio are sensitive to network size and degree structure. The degree-preserving null in Supplementary Table 4 conditions on each year’s observed network and therefore does not estimate a counterfactual in which network size is held constant across years. Accordingly, the longitudinal fragmentation results describe changes in the observed networks but cannot establish that fragmentation increased independently of network contraction. The category-level findings are less directly exposed to this concern because the attribute-permutation null holds each year’s network size and topology fixed while randomizing only actor labels (Supplementary Table 3). Fifth, part of the contraction coincided with the transition from the Historical PowerTrack API (2014–2019) to the Academic Research API (2020–2023). The defining core displacement occurred between 2014 and 2015, entirely within the continuous PowerTrack era, and the principal indicators are proportions rather than raw frequencies. Nonetheless, we cannot fully exclude residual differences in coverage between the two collection regimes.

The displacement of Health actors from the diffusion core and the presence of both Vape Community and Commercial accounts in the deepest diffusion core during four of the nine study years are empirical structural observations, but network topology alone cannot distinguish among three competing explanations: (1) coordinated inauthentic behavior, in which commercial or advocacy principals deliberately deploy human or automated agents to occupy diffusion backbones; (2) platform algorithmic sorting, in which engagement-maximizing content is systematically elevated regardless of actor intent; or (3) organic grassroots mobilization, in which motivated individuals independently converge on similar network positions through shared topical interest and community formation. Disentangling these mechanisms is a critical next step for research on structural fragility. Future work should integrate the structural indicators developed here with computational coordination detection (temporal burst analysis of posting synchronization, bot probability scoring of high-centrality accounts, and stylometric fingerprinting via transformer-based embeddings) to characterize not only whether public health communication is structurally marginalized, but how that marginalization arises, and thus which interventions are warranted.

## Supplementary information

Supplementary Information

## Data Availability

The Twitter/X data analyzed in this study cannot be shared in raw or post-level form. Per the data management and sharing plan submitted for this NIH-funded project (R01CA283038), data may only be shared and presented in aggregate form to avoid disclosing potentially identifiable information (e.g., Twitter/X usernames, individual tweet text). This restriction reflects privacy protections required by the study’s Institutional Review Board (IRB) exemption, applicable federal and state privacy laws, and contract terms and conditions governing Twitter/X API and content. The raw data are stored securely on restricted-access servers at NORC at the University of Chicago. Researchers may contact the Social Data Collaboratory at NORC at the University of Chicago (feng-miao@norc.org) for inquiries regarding data access. The Social Data Collaboratory will respond to inquiries as funding under the NIH grant project allows. Any shared data will be provided in aggregate form under a data use agreement prohibiting re-identification.

## Code availability

All network measures were computed in Python 3.14 using NetworkX 3.6.1, python-louvain 0.16, and python-igraph 1.0.0. The full structured prompt and classification schema for the automated account classification pipeline are provided in Supplementary Information. Custom code for network construction, metric computation, null-model analyses, trend tests, and figure generation is publicly available at https://github.com/Social-Data-Collaboratory/manuscript-structural-fragility-public and archived at https://zenodo.org/records/21983079.
