# Supplementary material for: Structural fragility in digital public health communication
Source: Commun Health. 2026 Oct 7;1(1):36. doi: 10.1038/s44528-026-00037-y (PMC13645615; doi:10.1038/s44528-026-00037-y)
Supplement: Supplementary file 1 [file 44528_2026_37_MOESM1_ESM.docx]

**Supplementary Information**

*Robustness and Null-Model Analyses*

*Retweet networks of e-cigarette prevention campaign discourse on Twitter/X, 2014–2023 (199,504 retweet events). Trend tests and null-model analyses use complete years 2014–2022 (nine annual observations); the partial 2023 period was retained for descriptive reporting only. Null models use 1,000 attribute permutations and 500 degree-preserving randomizations per year. Empirical p-values are two-sided.*

**Supplementary Table 1.** Monotonic trend tests (Mann–Kendall) and slopes (Theil–Sen) for fragility indicators, 2014–2022.

| Dimension | Indicator | Years | n | MK z | MK p | Theil-Sen slope/yr |
| --- | --- | --- | --- | --- | --- | --- |
| Community/topology | Louvain modularity | 2014-2022 | 9 | 2.189 | 0.0286 | 0.0481 |
| Community/topology | Largest connected component ratio | 2014-2022 | 9 | -3.232 | 0.0012 | -0.0513 |
| Community/topology | Deepest k-core depth (k*) | 2014-2022 | 9 | -0.738 | 0.4606 | -0.5500 |
| Community | Category assortativity | 2014-2022 | 9 | 2.189 | 0.0286 | 0.0812 |
| Community | Within-category retweet share | 2014-2022 | 9 | 2.189 | 0.0286 | 0.0401 |
| Core | Health share of deepest k-core | 2014-2022 | 9 | 0.000 | 1.0000 | 0.0036 |
| Core | Vape Community share of deepest k-core | 2014-2022 | 9 | 0.316 | 0.7518 | 0.0202 |
| Positional | Health share of eigenvector mass | 2014-2022 | 9 | 1.355 | 0.1753 | 0.0087 |
| Positional | Health share of PageRank mass | 2014-2022 | 9 | -0.313 | 0.7545 | -0.0055 |
| Positional | Health eigenvector Gini | 2014-2022 | 9 | 0.730 | 0.4655 | 0.0036 |
| Positional | Health eigenvector top-1% share | 2014-2022 | 9 | 1.147 | 0.2515 | 0.0290 |
| Positional | Health PageRank Gini | 2014-2022 | 9 | -2.398 | 0.0165 | -0.0497 |
| Positional | Health PageRank top-1% share | 2014-2022 | 9 | -2.398 | 0.0165 | -0.1035 |
| Positional | Health in-strength Gini | 2014-2022 | 9 | -3.232 | 0.0012 | -0.0147 |
| Positional | Health in-strength top-1% share | 2014-2022 | 9 | -2.815 | 0.0049 | -0.0831 |

***Note.*** *Mann–Kendall z statistics use tie-adjusted variance and the standard continuity correction. Mann–Kendall p-values are reported descriptively; see Methods regarding temporal dependence. Theil–Sen estimates represent median pairwise annual slopes. Top-1% shares are within-Health concentration measures. Core-fragility indicators are interpreted primarily through the attribute-permutation null because the 2014–2015 displacement is abrupt rather than monotonic.*

**Supplementary Table 2.** Inclusion-threshold sensitivity analysis: primary structural indicators under minimum activity thresholds of ≥1, ≥2, and ≥5 campaign-relevant retweets per user-year.

| Year | Min. retweets | Modularity | LCC ratio | Health k-core (%) | Vape Community k-core (%) | Organic node share (%) |
| --- | --- | --- | --- | --- | --- | --- |
| 2014 | ≥1 | 0.162 | 0.938 | 59.1 | 0.0 | 58.2 |
| 2014 | ≥2 | 0.201 | 0.951 | 59.1 | 0.0 | 56.0 |
| 2014 | ≥5 | 0.191 | 0.936 | 57.9 | 0.0 | 51.4 |
| 2015 | ≥1 | 0.662 | 0.951 | 3.8 | 78.8 | 53.3 |
| 2015 | ≥2 | 0.5 | 0.942 | 3.8 | 78.8 | 42.3 |
| 2015 | ≥5 | 0.4 | 0.964 | 3.8 | 78.8 | 34.3 |
| 2016 | ≥1 | 0.686 | 0.935 | 0.0 | 82.7 | 59.7 |
| 2016 | ≥2 | 0.610 | 0.967 | 0.0 | 82.7 | 48.2 |
| 2016 | ≥5 | 0.556 | 0.985 | 0.0 | 82.7 | 39.5 |
| 2017 | ≥1 | 0.53 | 0.897 | 3.7 | 0 | 71.9 |
| 2017 | ≥2 | 0.596 | 0.943 | 4.5 | 0 | 69.3 |
| 2017 | ≥5 | 0.564 | 0.942 | 4.5 | 0 | 60.3 |
| 2018 | ≥1 | 0.622 | 0.895 | 8.3 | 58.3 | 78.0 |
| 2018 | ≥2 | 0.632 | 0.944 | 8.3 | 58.3 | 77.7 |
| 2018 | ≥5 | 0.691 | 0.597 | 9.1 | 63.6 | 51.6 |
| 2019 | ≥1 | 0.785 | 0.819 | 34.5 | 27.7 | 57.8 |
| 2019 | ≥2 | 0.771 | 0.859 | 29.3 | 33.3 | 48.7 |
| 2019 | ≥5 | 0.718 | 0.627 | 35.5 | 25.8 | 51.8 |
| 2020 | ≥1 | 0.884 | 0.632 | 7.4 | 70.4 | 35.6 |
| 2020 | ≥2 | 0.838 | 0.816 | 4.5 | 68.2 | 19.9 |
| 2020 | ≥5 | 0.821 | 0.929 | 21.4 | 50.0 | 9.3 |
| 2021 | ≥1 | 0.771 | 0.677 | 3.7 | 70.4 | 38 |
| 2021 | ≥2 | 0.646 | 0.828 | 3.7 | 70.4 | 27.6 |
| 2021 | ≥5 | 0.517 | 0.962 | 3.7 | 70.4 | 11.4 |
| 2022 | ≥1 | 0.808 | 0.501 | 13.6 | 59.1 | 28.1 |
| 2022 | ≥2 | 0.714 | 0.699 | 13.6 | 59.1 | 22.5 |
| 2022 | ≥5 | 0.648 | 0.618 | 11.1 | 59.3 | 23.6 |

***Note.*** *Threshold denotes the number of campaign-relevant retweets made by each retweeter within a year; retweeted source accounts were retained as network endpoints. For modularity, values at the ≥1 threshold are taken directly from the primary analysis reported in main-text Table 2 and serve as the reference for within-table comparisons. Modularity at the ≥2 and ≥5 thresholds was computed on the corresponding weighted undirected projections using the Louvain algorithm with deterministic random seeds. The LCC ratio and k-core measures were recomputed directly for all thresholds and reproduced the Table 2 values exactly at ≥1. K-core composition and Organic node shares use the final account-level classifications.*

\

**Supplementary Table 3.** Attribute-permutation null model: observed category structure versus label-shuffled null (topology fixed; 1,000 permutations).

| Year | Indicator | Observed | Null mean | Null 95% lo | Null 95% hi | z | p |
| --- | --- | --- | --- | --- | --- | --- | --- |
| 2014 | Category assortativity | -0.4831 | -0.2518 | -0.3428 | -0.1787 | -3.175 | 0.0010 |
| 2015 | Category assortativity | -0.1590 | -0.0786 | -0.1720 | -0.0031 | -1.493 | 0.0759 |
| 2016 | Category assortativity | -0.0071 | -0.0567 | -0.1211 | -0.0149 | 1.468 | 0.1289 |
| 2017 | Category assortativity | -0.2708 | -0.0993 | -0.2627 | -0.0226 | -2.136 | 0.0100 |
| 2018 | Category assortativity | -0.3320 | -0.0882 | -0.2941 | 0.0026 | -2.697 | 0.0240 |
| 2019 | Category assortativity | -0.0886 | -0.0388 | -0.1084 | 0.0079 | -1.707 | 0.0819 |
| 2020 | Category assortativity | 0.1729 | -0.0106 | -0.0413 | 0.0220 | 11.446 | 0.0010 |
| 2021 | Category assortativity | 0.1016 | -0.0083 | -0.0399 | 0.0219 | 6.795 | 0.0010 |
| 2022 | Category assortativity | 0.2573 | -0.0062 | -0.0426 | 0.0301 | 14.192 | 0.0010 |
| 2014 | Health share of PageRank mass | 0.4101 | 0.0174 | 0.0062 | 0.0695 | 9.689 | 0.0010 |
| 2015 | Health share of PageRank mass | 0.3524 | 0.0188 | 0.0077 | 0.1380 | 9.263 | 0.0010 |
| 2016 | Health share of PageRank mass | 0.2312 | 0.0115 | 0.0040 | 0.0323 | 9.514 | 0.0010 |
| 2017 | Health share of PageRank mass | 0.3973 | 0.0136 | 0.0026 | 0.2983 | 6.615 | 0.0020 |
| 2018 | Health share of PageRank mass | 0.4408 | 0.0468 | 0.0082 | 0.4173 | 3.437 | 0.0020 |
| 2019 | Health share of PageRank mass | 0.3649 | 0.1087 | 0.0587 | 0.2578 | 4.440 | 0.0010 |
| 2020 | Health share of PageRank mass | 0.4248 | 0.2250 | 0.1277 | 0.4349 | 2.295 | 0.0400 |
| 2021 | Health share of PageRank mass | 0.2553 | 0.1402 | 0.0805 | 0.2435 | 2.640 | 0.0140 |
| 2022 | Health share of PageRank mass | 0.3444 | 0.2610 | 0.1865 | 0.3673 | 1.764 | 0.0739 |
| 2014 | Health share of deepest k-core | 0.5909 | 0.0184 | 0.000 | 0.0909 | 20.133 | 0.0010 |
| 2015 | Health share of deepest k-core | 0.0385 | 0.0179 | 0.000 | 0.0577 | 1.109 | 0.2438 |
| 2016 | Health share of deepest k-core | 0.000 | 0.0112 | 0.000 | 0.0385 | -0.760 | 0.6873 |
| 2017 | Health share of deepest k-core | 0.0370 | 0.0126 | 0.000 | 0.0741 | 1.101 | 0.2827 |
| 2018 | Health share of deepest k-core | 0.0833 | 0.0450 | 0.000 | 0.1667 | 0.620 | 1.000 |
| 2019 | Health share of deepest k-core | 0.3445 | 0.1056 | 0.0586 | 0.1597 | 8.652 | 0.0010 |
| 2020 | Health share of deepest k-core | 0.0741 | 0.2206 | 0.0741 | 0.4074 | -1.805 | 0.1019 |
| 2021 | Health share of deepest k-core | 0.0370 | 0.1382 | 0.0370 | 0.2593 | -1.498 | 0.1728 |
| 2022 | Health share of deepest k-core | 0.1364 | 0.2577 | 0.0909 | 0.4545 | -1.307 | 0.2228 |
| 2014 | Health share of eigenvector mass | 0.0287 | 0.0200 | 0.0185 | 0.0227 | 6.686 | 0.0050 |
| 2015 | Health share of eigenvector mass | 0.0147 | 0.0181 | 0.0028 | 0.0612 | -0.197 | 0.8621 |
| 2016 | Health share of eigenvector mass | 0.0017 | 0.0118 | 0.0000 | 0.0310 | -0.154 | 0.8751 |
| 2017 | Health share of eigenvector mass | 0.0254 | 0.0124 | 0.0095 | 0.0216 | 3.745 | 0.0160 |
| 2018 | Health share of eigenvector mass | 0.0199 | 0.0441 | 0.0387 | 0.0595 | -5.331 | 0.0010 |
| 2019 | Health share of eigenvector mass | 0.0420 | 0.1051 | 0.0825 | 0.1473 | -3.855 | 0.0010 |
| 2020 | Health share of eigenvector mass | 0.0160 | 0.2212 | 0.0026 | 0.8716 | -0.814 | 0.4925 |
| 2021 | Health share of eigenvector mass | 0.6621 | 0.1408 | 0.0316 | 0.3741 | 5.420 | 0.0010 |
| 2022 | Health share of eigenvector mass | 0.5120 | 0.2609 | 0.0651 | 0.5569 | 1.814 | 0.0490 |
| 2014 | Vape Community share of deepest k-core | 0.000 | 0.0023 | 0.0000 | 0.0455 | -0.220 | 1.0000 |
| 2015 | Vape Community share of deepest k-core | 0.7885 | 0.0312 | 0.0000 | 0.0769 | 31.874 | 0.0010 |
| 2016 | Vape Community share of deepest k-core | 0.8269 | 0.0599 | 0.0000 | 0.1346 | 23.967 | 0.0010 |
| 2017 | Vape Community share of deepest k-core | 0.000 | 0.0079 | 0.0000 | 0.0370 | -0.476 | 1.0000 |
| 2018 | Vape Community share of deepest k-core | 0.5833 | 0.0116 | 0.0000 | 0.0833 | 18.756 | 0.0010 |
| 2019 | Vape Community share of deepest k-core | 0.2773 | 0.0384 | 0.0084 | 0.0756 | 13.222 | 0.0010 |
| 2020 | Vape Community share of deepest k-core | 0.7037 | 0.1422 | 0.0370 | 0.2963 | 8.148 | 0.0010 |
| 2021 | Vape Community share of deepest k-core | 0.7037 | 0.1580 | 0.0370 | 0.2963 | 7.465 | 0.0010 |
| 2022 | Vape Community share of deepest k-core | 0.5909 | 0.1466 | 0.0000 | 0.3182 | 5.927 | 0.0010 |
| 2014 | Within-category retweet share | 0.0674 | 0.4927 | 0.3960 | 0.5799 | -4.071 | 0.0210 |
| 2015 | Within-category retweet share | 0.2541 | 0.4453 | 0.2780 | 0.5139 | -3.266 | 0.0050 |
| 2016 | Within-category retweet share | 0.2994 | 0.4448 | 0.2688 | 0.5759 | -1.770 | 0.0709 |
| 2017 | Within-category retweet share | 0.1697 | 0.5888 | 0.2384 | 0.7022 | -2.889 | 0.0010 |
| 2018 | Within-category retweet share | 0.1534 | 0.6353 | 0.2496 | 0.7695 | -2.975 | 0.0010 |
| 2019 | Within-category retweet share | 0.2582 | 0.4293 | 0.2532 | 0.5291 | -2.062 | 0.0320 |
| 2020 | Within-category retweet share | 0.4219 | 0.2820 | 0.2357 | 0.3313 | 5.493 | 0.0010 |
| 2021 | Within-category retweet share | 0.3844 | 0.2943 | 0.2462 | 0.3422 | 3.610 | 0.0020 |
| 2022 | Within-category retweet share | 0.4265 | 0.2669 | 0.2264 | 0.3097 | 7.550 | 0.0010 |

***Note.*** *Category labels permuted uniformly at random across nodes within each yearly network (1,000 permutations); topology and category counts preserved. Two-sided empirical p-values based on absolute deviation from the null mean; no multiple-comparison adjustment across years or indicators. Category assortativity is computed on the unweighted undirected projection (matching the main analysis); within-category retweet share is event-weighted on the directed graph. 2023 (partial year) excluded.*

**Supplementary Table 4.** Degree-preserving configuration-model null: observed global topology versus degree-matched random networks.

| Year | Indicator | Observed | Null mean | Null 95% lo | Null 95% hi | z | p |
| --- | --- | --- | --- | --- | --- | --- | --- |
| 2014 | Deepest k-core (k_max) | 4.0000 | 5.4980 | 5.0000 | 6.0000 | -2.8 | 0.0200 |
| 2015 | Deepest k-core (k_max) | 24.0000 | 21.3500 | 21.0000 | 22.0000 | 4.9 | 0.0020 |
| 2016 | Deepest k-core (k_max) | 27.0000 | 22.1340 | 21.0000 | 23.0000 | 7.8 | 0.0020 |
| 2017 | Deepest k-core (k_max) | 8.0000 | 11.4180 | 11.0000 | 12.0000 | -6.2 | 0.0020 |
| 2018 | Deepest k-core (k_max) | 5.0000 | 6.8700 | 6.0000 | 8.0000 | -3.9 | 0.0020 |
| 2019 | Deepest k-core (k_max) | 3.0000 | 6.6880 | 6.0000 | 7.0000 | -7.6 | 0.0020 |
| 2020 | Deepest k-core (k_max) | 4.0000 | 5.3500 | 5.0000 | 6.0000 | -2.8 | 0.0060 |
| 2021 | Deepest k-core (k_max) | 6.0000 | 7.4860 | 7.0000 | 8.0000 | -2.9 | 0.0040 |
| 2022 | Deepest k-core (k_max) | 5.0000 | 4.9620 | 4.0000 | 6.0000 | 0.1 | 1.0000 |
| 2014 | LCC ratio | 0.9380 | 0.9429 | 0.9415 | 0.9443 | -6.6 | 0.0020 |
| 2015 | LCC ratio | 0.9508 | 0.9608 | 0.9580 | 0.9637 | -6.8 | 0.0020 |
| 2016 | LCC ratio | 0.9347 | 0.9123 | 0.9077 | 0.9169 | 9.9 | 0.0020 |
| 2017 | LCC ratio | 0.8968 | 0.8879 | 0.8841 | 0.8915 | 4.7 | 0.0020 |
| 2018 | LCC ratio | 0.8947 | 0.8862 | 0.8824 | 0.8903 | 4.0 | 0.0020 |
| 2019 | LCC ratio | 0.8186 | 0.7506 | 0.7402 | 0.7613 | 12.8 | 0.0020 |
| 2020 | LCC ratio | 0.6319 | 0.7001 | 0.6757 | 0.7233 | -5.7 | 0.0020 |
| 2021 | LCC ratio | 0.6766 | 0.7508 | 0.7240 | 0.7793 | -5.2 | 0.0020 |
| 2022 | LCC ratio | 0.5012 | 0.7561 | 0.7262 | 0.7869 | -15.2 | 0.0020 |
| 2014 | Louvain modularity | 0.1639 | 0.1682 | 0.1631 | 0.1751 | -1.4 | 0.1597 |
| 2015 | Louvain modularity | 0.6604 | 0.5933 | 0.5852 | 0.6020 | 15.3 | 0.0020 |
| 2016 | Louvain modularity | 0.6823 | 0.6200 | 0.5734 | 0.6656 | 1.8 | 0.0020 |
| 2017 | Louvain modularity | 0.5327 | 0.5085 | 0.4929 | 0.5246 | 2.9 | 0.0040 |
| 2018 | Louvain modularity | 0.6233 | 0.5438 | 0.5320 | 0.5554 | 13.4 | 0.0020 |
| 2019 | Louvain modularity | 0.7864 | 0.7378 | 0.7249 | 0.7489 | 7.9 | 0.0020 |
| 2020 | Louvain modularity | 0.8860 | 0.7862 | 0.7716 | 0.7974 | 14.3 | 0.0020 |
| 2021 | Louvain modularity | 0.7670 | 0.7351 | 0.7226 | 0.7482 | 4.8 | 0.0020 |
| 2022 | Louvain modularity | 0.8100 | 0.7396 | 0.7277 | 0.7524 | 11.2 | 0.0020 |

***Note.*** *Networks were randomized using degree-preserving simple-graph rewiring with 500 randomizations per year; the observed edge-weight multiset was randomly reassigned to rewired edges. Empirical p-values are two-sided and based on absolute deviation from the null mean; no multiple-comparison adjustment was applied across years or indicators. Analyses use fixed random seeds and reproduce exactly under the software versions reported in Code Availability. For internal consistency within this null-model analysis, modularity for both observed and randomized networks was computed using igraph's multilevel algorithm. Main-text modularity values were computed using python-louvain on the same undirected weighted graph projection. Annual observed modularity values differ by less than 0.005 between the two implementations and yield the same substantive interpretation. LCC ratio and maximum k-core depth do not depend on the community-detection implementation and reproduce the corresponding main-analysis values.*

**Supplementary Table 5.** Descriptive network statistics for health actors, 2014 – 2023*

|  | 2014 | 2015 | 2016 | 2017 | 2018 | 2019 | 2020 | 2021 | 2022 | 2023* |
| --- | --- | --- | --- | --- | --- | --- | --- | --- | --- | --- |
| Accounts (n) | 727 | 392 | 243 | 265 | 569 | 600 | 317 | 168 | 210 | 47 |
| In-strength share | 0.93 | 0.58 | 0.36 | 0.66 | 0.62 | 0.69 | 0.53 | 0.28 | 0.44 | - |
| Total in-degree per 1,000 members | 45,380 | 47,702 | 56,214 | 56,879 | 16,271 | 6,592 | 2,467 | 2,494 | 1,957 | - |
| Accounts retweeted by ≥1 other account (%) | 13.2 | 14.8 | 24.7 | 14.0 | 18.8 | 27.7 | 34.4 | 36.3 | 35.2 | - |
| PageRank share | 0.41 | 0.35 | 0.23 | 0.40 | 0.44 | 0.36 | 0.42 | 0.26 | 0.34 | - |
| Deepest k-core share (%) | 59.1 | 3.8 | 0.0 | 3.7 | 8.3 | 34.5 | 7.4 | 3.7 | 13.6 | - |

*Note: *January–April 2023; partial-year values are descriptive.*

**Supplementary Table 6.** Descriptive network statistics for commercial accounts, 2014 – 2023*

|  | 2014 | 2015 | 2016 | 2017 | 2018 | 2019 | 2020 | 2021 | 2022 | 2023* |
| --- | --- | --- | --- | --- | --- | --- | --- | --- | --- | --- |
| Accounts (n) | 50 | 392 | 891 | 161 | 66 | 61 | 34 | 19 | 16 | 5 |
| In-strength share | 0.00 | 0.02 | 0.15 | 0.01 | 0.01 | 0.02 | 0.09 | 0.01 | 0.04 | - |
| Total in-degree per 1,000 members | 40 | 1,712 | 2,349 | 932 | 606 | 1,197 | 1,971 | 1,316 | 1,312 | - |
| Accounts retweeted by ≥1 other account (%) | 2.0 | 16.6 | 12.8 | 18.0 | 18.2 | 26.2 | 29.4 | 21.1 | 31.2 | - |
| PageRank share | 0.00 | 0.01 | 0.04 | 0.00 | 0.00 | 0.01 | 0.02 | 0.01 | 0.02 | - |
| Deepest k-core share (%) | 4.5 | 7.7 | 7.7 | 0.0 | 0.0 | 4.2 | 7.4 | 0.0 | 0.0 | - |

*Note: *January–April 2023; partial-year values are descriptive.*

**Supplementary Table 7.** Descriptive network statistics for vape community accounts, 2014 – 2023*

|  | 2014 | 2015 | 2016 | 2017 | 2018 | 2019 | 2020 | 2021 | 2022 | 2023* |
| --- | --- | --- | --- | --- | --- | --- | --- | --- | --- | --- |
| Accounts (n) | 88 | 681 | 1,298 | 188 | 129 | 213 | 203 | 194 | 116 | 24 |
| In-strength share | 0.00 | 0.30 | 0.31 | 0.01 | 0.03 | 0.05 | 0.20 | 0.32 | 0.19 | - |
| Total in-degree per 1,000 members | 102 | 6,283 | 4,308 | 1,394 | 1,977 | 1,779 | 1,951 | 3,057 | 1,707 | - |
| Accounts retweeted by ≥1 other account (%) | 6.8 | 30.1 | 16.6 | 18.6 | 17.1 | 30.5 | 29.1 | 33.5 | 30.2 | - |
| PageRank share | 0.00 | 0.07 | 0.12 | 0.01 | 0.01 | 0.04 | 0.12 | 0.20 | 0.14 | - |
| Deepest k-core share (%) | 0.0 | 78.8 | 82.7 | 0.0 | 58.3 | 27.7 | 70.4 | 70.4 | 59.1 | - |

*Note: *January–April 2023; partial-year values are descriptive.*

**Supplementary Table 8.** Descriptive network statistics for organic accounts, 2014 – 2023*

|  | 2014 | 2015 | 2016 | 2017 | 2018 | 2019 | 2020 | 2021 | 2022 | 2023* |
| --- | --- | --- | --- | --- | --- | --- | --- | --- | --- | --- |
| Accounts (n) | 21,066 | 11,436 | 12,651 | 15,374 | 10,076 | 3,310 | 513 | 461 | 225 | 25 |
| In-strength share | 0.04 | 0.04 | 0.06 | 0.14 | 0.06 | 0.12 | 0.06 | 0.03 | 0.09 | - |
| Total in-degree per 1,000 members | 72 | 123 | 182 | 212 | 91 | 227 | 234 | 106 | 444 | - |
| Accounts retweeted by ≥1 other account (%) | 3.3 | 4.1 | 5.1 | 5.8 | 3.8 | 12.4 | 9.9 | 6.3 | 14.2 | - |
| PageRank share | 0.31 | 0.25 | 0.26 | 0.20 | 0.15 | 0.30 | 0.18 | 0.17 | 0.16 | - |
| Deepest k-core share (%) | 9.1 | 5.8 | 5.8 | 55.6 | 25.0 | 13.4 | 3.7 | 0.0 | 13.6 | - |

*Note: *January–April 2023; partial-year values are descriptive.*

**Supplementary Table 9.** Descriptive network statistics for Other accounts, 2014 – 2023*

|  | 2014 | 2015 | 2016 | 2017 | 2018 | 2019 | 2020 | 2021 | 2022 | 2023* |
| --- | --- | --- | --- | --- | --- | --- | --- | --- | --- | --- |
| Accounts (n) | 14,273 | 8,537 | 6,097 | 5,397 | 2,085 | 1,539 | 373 | 370 | 233 | 21 |
| In-strength share | 0.03 | 0.06 | 0.12 | 0.18 | 0.27 | 0.11 | 0.12 | 0.36 | 0.24 | - |
| Total in-degree per 1,000 members | 80 | 147 | 656 | 706 | 1,222 | 404 | 499 | 1,108 | 1,030 | - |
| Accounts retweeted by ≥1 other account (%) | 3.6 | 3.2 | 7.4 | 9.9 | 6.7 | 11.6 | 8.3 | 11.6 | 15.5 | - |
| PageRank share | 0.28 | 0.31 | 0.34 | 0.39 | 0.40 | 0.28 | 0.25 | 0.36 | 0.33 | - |
| Deepest k-core share (%) | 27.3 | 3.8 | 3.8 | 40.7 | 8.3 | 20.2 | 11.1 | 25.9 | 13.6 | - |

*Note: *January–April 2023; partial-year values are descriptive.*

*Additional Methodological Details*

**Supplementary Table 10.** GPT-4o Account-Classification Performance by Class

| Class | Precision | Recall | F1 Score | Support (N Users) |
| --- | --- | --- | --- | --- |
| Organic | 0.95 | 0.79 | 0.86 | 48 |
| Health | 0.95 | 0.95 | 0.95 | 40 |
| Commercial | 0.88 | 0.83 | 0.85 | 42 |
| Vape Community | 0.75 | 0.86 | 0.80 | 35 |
| Other | 0.78 | 0.89 | 0.83 | 35 |

*Note. Support counts reflect adjudicated human-reference labels in the 200-account validation sample. Because sampling was stratified by GPT-4o-assigned category, adjudicated class counts need not equal 40 in every class.*

**Labeling Codebook for Human Account Classification**

*Codebook used by two trained coders to assign account-level labels to the 160-account reliability sample and the 200-account validation sample; disagreements were adjudicated by a senior coder. Category labels shown here are the production labels used during coding.*

**General Instructions & Principles:**

**1**. **Goal:** Assign each unique Twitter/X user account relevant to the study into **one and only one primary category** from the five defined below (Health Actor [Health], Commercial, Vape Community, Organic User [Organic], and Other).

**2.** **Mutual Exclusivity:** These categories are designed to be mutually exclusive based on the account’s primary function, identity, and publicly presented information.

**3.** **Data Sources:** Classification should be based primarily on **Username, Actor Name, Actor Summary/Bio, and/or Profile Picture**. Reference recent tweets (max 10) *only* if needed to clarify primary function or resolve ambiguity. If bio is missing/different via Twitter/X link, refer to the archived profile summary field. Note that the GPT-4o prompt supplied a single example tweet; human coders could therefore consult somewhat richer evidence when resolving ambiguous cases.

**4.** **Decision Hierarchy:** For accounts that potentially exhibit characteristics of multiple categories, classification should follow the **decision rules and hierarchy outlined below**. (Example priority: An explicit Commercial selling role generally overrides a Vape Community role if performed by the same entity; a clear Health Actor role overrides Organic if discussing health professionally).

**Category Definitions:**

**(1) Health Actor (Institutions & Affiliated Individuals)**

**INCLUSION:**

- Official accounts of health-related organizations or individuals including public health agencies, healthcare providers, researchers, etc. (healthcare organizations, governmental like FDA, CDC, state/local health depts; non-profits like Campaign for Tobacco-Free Kids, Truth Initiative).
- Accounts of individuals clearly identifiable (by bio, username, linked website) as healthcare professionals (Dr., RN, etc.), public health researchers/scientists, or employees of health organizations*.*
- Bio/username explicitly mentions relevant health roles or links to established health organization websites.

**EXCLUSION:** Accounts primarily fitting Commercial or Vape Community categories; Organic users not discussing health in a professional capacity; general news outlets.

*Relevant Terms Hint:* health, public health, Dr., MD, MPH, PhD (in relevant field), nurse, RN, physician, dentist, psychiatrist, psychologist, epidemiologist, researcher, scientist, surgeon, health dept, CDC, FDA, NIH, non-profit health org names.

**(2) Commercial**

**INCLUSION:**

- Accounts of organizations, brands, or individuals whose primary function (based on profile, name, main activity) is explicitly **selling or promoting e-cigarette/vaping products, liquids, or related accessories**. (e.g., vape shops, online retailers, manufacturers, brand-specific accounts).
- Profile contains direct links to commercial websites selling these products.
- Frequent posts about deals, sales, new products, or direct product marketing.

**EXCLUSION:** Accounts whose primary role is advocacy, even if they support commercial interests (these go to Vape Community); organic users occasionally mentioning products they use; general retailers not specializing in vape products.

**Decision Rule:** If an account sells products AND advocates, classify as **Commercial** if selling appears to be the primary business/identity.

*Relevant Terms Hint:* vape shop, e-liquid, e-juice, wholesale, retail, buy vape, deals, discount, brand names (like JUUL, Vuse - *if it's the official brand account*), vape mods, coils, tanks.

**(3) Vape Community**

**INCLUSION:**

- Accounts of organizations or individuals whose **primary function** is **advocating for vaping use, promoting pro-vaping policies/rights, arguing against vaping regulations, or actively opposing/countering public health prevention campaigns/messages**, *without* primarily being a commercial seller.
- Profile explicitly states advocacy mission (e.g., vapers’ rights, harm reduction advocate, fighting vape bans).
- Frequent use of Vape Community hashtags/slogans (see list below).
- Promotes pro-vaping organizations (e.g., CASAA) or media (e.g., A Billion Lives).
- Content focuses on political action, testimonials *for advocacy purposes*, critiquing public health/science perceived as anti-vaping.
- Includes accounts whose primary function is opposing or disrupting prevention campaigns.

**EXCLUSION:** Accounts whose primary function is Commercial selling; Health Actors; Organic users merely expressing personal enjoyment of vaping or describing being a vaper without broader advocacy aims.

*Relevant Terms Hint:* harm reduction advoc*, tobacco harm reduction, vap* advoc*, e-cig* advoc*, vaping rights, consumer advocates, CASAA, #vapelyfe, #ABillionLives, #ivapeivote, #notblowingsmoke, #adultslikeflavors, #vapingsaveslives. *Check website links if provided.*

**(4) Organic User**

**INCLUSION:**

- Accounts clearly identifiable as **private individuals** based on profile picture (individual person), name usage (first/last name), bio details (personal description, non-professional job, age, location, pronouns).
- AND **do not primarily fit** the definitions of Health Actor, Commercial, or Vape Community based on their profile/bio related to the topic.
- May express personal opinions about vaping (pro or con), share news, or interact casually without representing an organization or a dedicated vape community/commercial role.

**EXCLUSION:** Accounts meeting criteria for Health, Commercial, or Vape Community; organization/brand accounts; prominent public figures (e.g., >10k followers unless clearly fitting another category); community/fan accounts for specific topics (unless advocacy); meme accounts; accounts lacking sufficient info (go to Other).

**Default Rule Clarification:** Code as Organic only if clear individual cues are present AND they *don’t* meet primary criteria for the other specific categories.

**(5) Other / Unclassifiable**

**INCLUSION:**

- Accounts that cannot be confidently assigned to any of the above four categories based on available profile information.
- Accounts with insufficient information (no bio, no identifying pic/name, protected tweets if profile inaccessible).

**Structured Prompt Template and Output Schema Used for GPT-4o Account Classification**

SYSTEM

You are a careful annotator. Review each Twitter/X user profile and RETURN a single JSON object assigning the account to exactly one category. Follow definitions and edge cases exactly. Do NOT output prose.

RULES

- Treat each account independently.

- Base classification on all provided fields (profile info, bio/summary, follower/friend counts, and sample post).

- Be conservative: only assign a category if clearly supported.

- If uncertain, assign "Other."

- Return exactly one category, always valid JSON.

FIELDS PROVIDED

- actorname: Display name

- actorpreferredusername: Twitter/X handle

- actorsummary: Profile bio/description

- actorfollowersCount: Followers count

- actorfriendsCount: Following count

- actorfavoritesCount: Likes given

- actorstatusuesCount: Posts made

- actorlistedCount: Public list inclusions

- actorverified: Verification status

- bodypost: Example tweet/post text

CATEGORIES^[1]^

1) Public Health Actor: Official health orgs or identifiable professionals engaging on tobacco/e-cigarette issues.

2) Commercial: Accounts selling or promoting e-cigarette/vaping products or brands.

3) Vape Community: Advocates for vaping (policies/rights), not primarily sellers.

4) Organic User: Private individuals (personal bio, name, age, pronouns), not fitting above categories.

5) Other: Insufficient info to confidently classify.

OUTPUT SCHEMA

{

 "category": "Public Health Actor" | "Commercial" | "Vape Community" | "Organic User" | "Other"

}

INPUT FORMAT (JSON of relevant fields)

{

 "actorname": "...",

 "actorpreferredusername": "...",

 "actorsummary": "...",

 "actorfollowersCount": ...,

 "actorfriendsCount": ...,

 "actorfavoritesCount": ...,

 "actorstatusuesCount": ...,

 "actorlistedCount": ...,

 "actorverified": "...",

 "bodypost": "..."

}

RESPONSE FORMAT

- Output only the JSON object above. No extra text, no comments, no prose.

**Supplementary Figure 1. Data flow diagram for analytic tweet selection.**

1. The production prompt used the output labels “Public Health Actor” and “Organic User.” These were harmonized to “Health” and “Organic,” respectively, in the analytic dataset; the remaining category labels were unchanged.
